# Altered Intrinsic Connectivity Networks in Frontal Lobe Epilepsy: A Resting-State fMRI Study

**DOI:** 10.1155/2014/864979

**Published:** 2014-11-26

**Authors:** Xinzhi Cao, Zhiyu Qian, Qiang Xu, Junshu Shen, Zhiqiang Zhang, Guangming Lu

**Affiliations:** ^1^Department of Biomedical Engineering, Nanjing University of Aeronautics and Astronautics, Nanjing 210016, China; ^2^Department of Medical Imaging, Jinling Hospital, Medical School of Nanjing University, Nanjing 210002, China; ^3^Department of Radiotherapy, Jinling Hospital, Medical School of Nanjing University, Nanjing 210002, China

## Abstract

Examining the resting-state networks (RSNs) may help us to understand the neural mechanism of the frontal lobe epilepsy (FLE). Resting-state functional MRI (fMRI) data were acquired from 46 patients with FLE (study group) and 46 age- and gender-matched healthy subjects (control group). The independent component analysis (ICA) method was used to identify RSNs from each group. Compared with the healthy subjects, decreased functional connectivity was observed in all the networks; however, in some areas of RSNs, functional connectivity was increased in patients with FLE. The duration of epilepsy and the seizure frequency were used to analyze correlation with the regions of interest (ROIs) in the nine RSNs to determine their influence on FLE. The functional network connectivity (FNC) was used to study the impact on the disturbance and reorganization of FLE. The results of this study may offer new insight into the neuropathophysiological mechanisms of FLE.

## 1. Introduction

As the second highest type of localization-related epilepsies, frontal lobe epilepsy (FLE) accounts for 20 to 30 percent among all partial epilepsies [1]. However, compared with temporal lobe epilepsy (TLE), FLE in general has been less studied. Seizures in patients with FLE are varied and can be divided into perirolandic, supplementary sensorimotor area, dorsolateral, orbitofrontal, anterior frontopolar, opercular, and cingulate types [2]. Seizures originating from the perirolandic and supplementary sensorimotor areas can be distinguished, by the manifestation of motor activity or asymmetric tonic posturing with preserved awareness. However, seizures arising from dorsolateral, orbitofrontal, frontopolar, and cingulate areas have more variable clinical manifestations, and they cannot be distinguished from each other easily. The seizures frequently occur during sleep, without warning, are very short, and are followed by a rapid recovery [3].

Except for the obvious abnormalities, such as previous brain trauma, neoplasms, vascular malformations, and developmental lesions, no clear cause has been found in most patients with FLE. Furthermore, regular structure MRI imaging shows nothing valuable. However, the mechanism of how the epilepsy focus influences the functional network and further leads to the deficits of the brain's function and cognitive behavior is still not clear. Contemporary fMRI has provided effective approaches for measuring the functional alteration caused by epilepsy in brain, which is a four-dimensional medical imaging modality that captures changes in blood oxygenation over time, an indirect measure of neuronal activation.

Although EEG-fMRI and resting-state fMRI are commonly used in the study of epilepsy [4, 5], scalp recording of EEG changes in FLE often had the poor spatial resolution and discharge localization [6]. Resting-state fMRI is a powerful technique for the exploring of abnormal intrinsic activity caused by epilepsy in the brain, with good spatial and temporal resolution. ICA is a powerful method to investigate the resting-state fMRI data. As a blind source separation method, it can recover a set of signals from their linear mixtures and has yielded fruitful results with fMRI data [7]. Spatial ICA decomposes fMRI data into a set of maximally spatially independent maps and their corresponding time courses [8]. The dynamics of the blood oxygen level dependent (BOLD) signal within a single component is described by that component's time courses in resting-state fMRI data. Regions contributing significantly within a given component are strongly functionally connected to each other. This kind of biological relevance is known as resting-state networks (RSNs) [9]. RSNs are used as a robust tool to the intrinsic functional architecture of the human brain for their high reproducibility and moderate to high test-retest reliability [10].

Most studies have focused on the RSNs used with resting-state fMRI in epilepsy. Widjaja et al. showed impaired default mode network (DMN) in children with medically refractory epilepsy [11]. Zhang et al. found dorsal attention network (DAN) and auditory network (AN) impaired in temporal lobe epilepsy (TLE) [12, 13]. Woodward et al. considered functional connectivity decreased in the motor network [14], and they all concluded that the motor network had been impaired. Wang et al. found functional connectivity decreased in the self-referential network (SRN), somatomotor network (SMN), and visual network (VN); they also found both decreased and increased in the DMN and DAN in patients with generalized tonic-clonic seizures (GTCS) [15]. Luo et al. presented reduction of functional connectivity in SMN when using group ICA on resting-state fMRI data [16].

Amann et al. extracted ROIs, regions of altered functional connectivity in brain, from RSNs and used *z*-scores obtained from ICA analysis for correlation analysis [17]. Braakman et al. found the ROIs of the RSNs were related to epilepsy factors including duration of epilepsy and seizure frequency [18]. These results may be useful to understand the influence of epilepsy with those clinical variables.

However, these studies were all focused on single resting-state functional network. Little attention was paid to the interactions between the RSNs in patients with epilepsy. Luo et al. implicated that investigating the interactions between the RSNs might be helpful to understand the neuropathophysiological mechanism of epilepsy globally [16]. By using functional network connectivity (FNC), they found that the lost interactions among the intersystem might be associated with the disturbance of the high level complex function which is needed in the integrated multisystems of partial epilepsy. The FNC toolbox calculated the time lags between components correlated with each other, and FNC maps were constructed via a partial correlation analysis of ICA time courses. This approach has been published and applied to the identification of which pairs of RSNs show changes in temporal correlation between patients and healthy controls (HC) [19].

FLE may involve multiple interacting networks, and few studies have focused on analyzing the functional connectivity of RSNs in patients with FLE and FNC relationships. Investigations on RSNs in patients with FLE could provide valuable data to validate the hypothesis that the abnormal neuroactivity generated from epilepsy focus leads to functional network impairment. The primary aim of the present work was to determine whether the intranetwork of RSNs might be aberrant in patients with FLE on resting-state fMRI data. The study also was concerned about the significant correlation between altered ROIs of the RSNs and clinical variables, including seizure frequency and the duration of epilepsy. Examination of the differences in FNC between the patients with FLE and HC may be helpful to understand the impact on the disturbance and reorganization of FLE. The results of this study may provide new insight into the neuropathophysiological mechanisms of FLE.

## 2. Material and Methods

### 2.1. Participants

Patients with FLE were recruited from a patient population who had received clinical treatments in Jinling Hospital. Forty-six healthy subjects were selected as the control group that matches the age and gender of the FLE group. Two-sample *t*-test of age showed *P* > 0.05, and the result of Pearson chi-square test of gender showed *P* > 0.05. Clinical information and demographic of patients with FLE and HC are shown in Table 1.

Patients with FLE were diagnosed based on the International League against Epilepsy (ILAE) classification [20]. Moreover, all the patients reached the inclusive criteria as follows: (1) one or more typical symptoms of FLE, including night seizure or posturing; (2) during electrophysiological monitoring, ictal EEG demonstrated typical generalized epileptiform spikes; (3) predominantly interictal epileptic discharges were shown by scalp EEGs; (4) there was no abnormality in the images of regular structural MRI. All subjects gave their written informed consent to participate in this study, which had been approved by the Medical Ethics Committee in Jinling Hospital.

### 2.2. Data Acquisition

Functional MRI data were acquired on a Magnetom Trio 3T MR Scanner (Siemens AG, Erlangen, Germany). During the resting-state fMRI session, the participants were instructed to relax with their eyes closed and keep their heads still during the scans. Functional images were subsequently acquired in the same slice orientation with a GRE-EPI (gradient recalled echo, echo-planar imaging) sequence (TR/TE = 2,000 ms/30 ms, FOV 24.0 × 24.0 cm^2^, FA 90°, matrix 64 × 64, slice thickness 4.0 mm, slice gap 0.4 mm, 30 slices, acquisition voxel size = 3.0 × 3.0 × 3.0 mm) for eight minutes and twenty seconds (250 measurements). 3D T1-weighted images were also acquired by using a 3DMPRAGE sequence, matrix 256 × 256, slice thickness 1.0 mm.

### 2.3. fMRI Data Preprocessing

A SPM- (statistical parametric mapping-) based fMRI data processing pipeline DPARSF (http://www.restfmri.net/forum/) was used to perform the data preprocessing, including the slice timing correction and motion correction. Data was excluded if the head motion exceeded 1.0 mm or if rotation exceeded 1.0° during scanning. The 3D T1-weighted images were used for normalizing the fMRI images after being resampled to 3.0 mm × 3.0 mm × 3.0 mm. After being smoothed with a full width at half maximum (FWHM) of 8.0 mm, the fMRI data were prepared for further processing.

### 2.4. RSNs Extraction Using ICA

**Figure 1:**
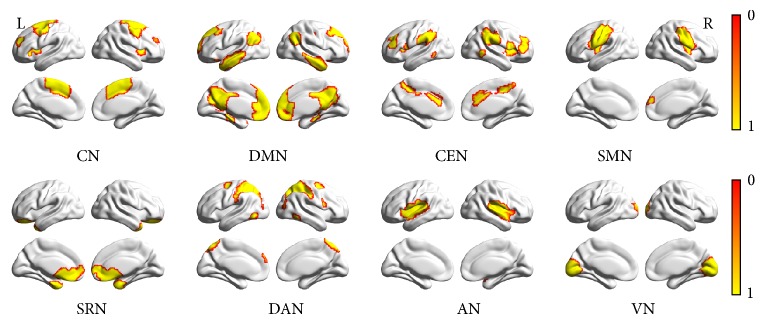
Eight templates of RSNs for estimating maximally independent spatial components among ninety-two subjects, including CN, DMN, CEN, SMN, SRN, DAN, AN, and VN, shown by BrainNet Viewer. The *T* value range is 0-1.

Group spatial ICA was conducted by using the infomax algorithm with the GIFT software (http://icatb.sourceforge.net/) in Matlab (The MathWorks Inc.). The fMRI data of the FLE and HC group were calculated to identify intrinsic networks, by estimating maximally independent spatial sources among ninety-two subjects. The data of all 92 subjects were decomposed into 29 independent components (ICs). As a preprocessing step to ICA decomposition, the principal component analysis (PCA) was conducted to reduce data dimensions. Single subject time courses and spatial maps were then back-reconstructed. The relatively high model order ICA was chosen, which could yield refined components [21]. The eight ICs for further analysis were selected based on the largest spatial correlation [22] with specific RSN templates reported in previous studies [23–25], which correspond to known anatomical and functional segmentations. The RSN templates are shown in Figure 1. These were done by using BrainNet Viewer [26] and included core network (CN), default mode network (anterior DMN, posterior DMN), central executive network (CEN), somatomotor network (SMN), self-referential network (SRN), dorsal attention network (DAN), auditory network (AN), and visual network (VN).

### 2.5. Intranetwork Analysis

Group comparisons were restricted to the voxels within each corresponding RSN. Two-sample *t*-test (*P* < 0.05, AlphaSim corrected) was employed for group comparisons for the areas within the mask of each RSN. The mask for group comparisons was created by combining the regions of the RSN by using one-sample *t*-test (*P* < 0.05, AlphaSim corrected) [27] among the ICs marked as the same RSNs of each individual subject in both the patients and controls [28].

The effects of clinical variables including seizure frequency and the duration of epilepsy were analyzed in correlation with the alterations in RSNs in epilepsy. From each corresponding RSN, the voxels showing significant differences (positive or negative) between the patient and the control groups were extracted as a mask consisting of several regions of interest (ROIs). The mean *z*-scores, obtained from ICA analysis, within each mask were correlated with the seizure frequency and the duration of epilepsy, which is considered to reflect the influence of the disease.

### 2.6. Internetwork Analysis

The ICA algorithm assumed that the time courses of cortical areas within one component were synchronous. Though the components were spatially independent, significant temporal correlations could exist between them. As an extension of ICA analysis, the FNC toolbox (http://mialab.mrn.org/software/#fnc) was employed to examine the temporal relationships between brain networks. Corresponding to the significant correlation combinations, the average time lags, which represented the amount of delay between two correlated RSNs' time courses, were calculated for each group. The maximum time lag was set to 3 s [29]. One-sample *t*-test (*P* < 0.05, FDR corrected) for each group and two-sample *t*-test (*P* < 0.05, FDR corrected) for group comparisons were performed on all possible combinations [16].

## 3. Results

### 3.1. Intranetwork Alterations of RSNs in FLE

**Figure 2:**
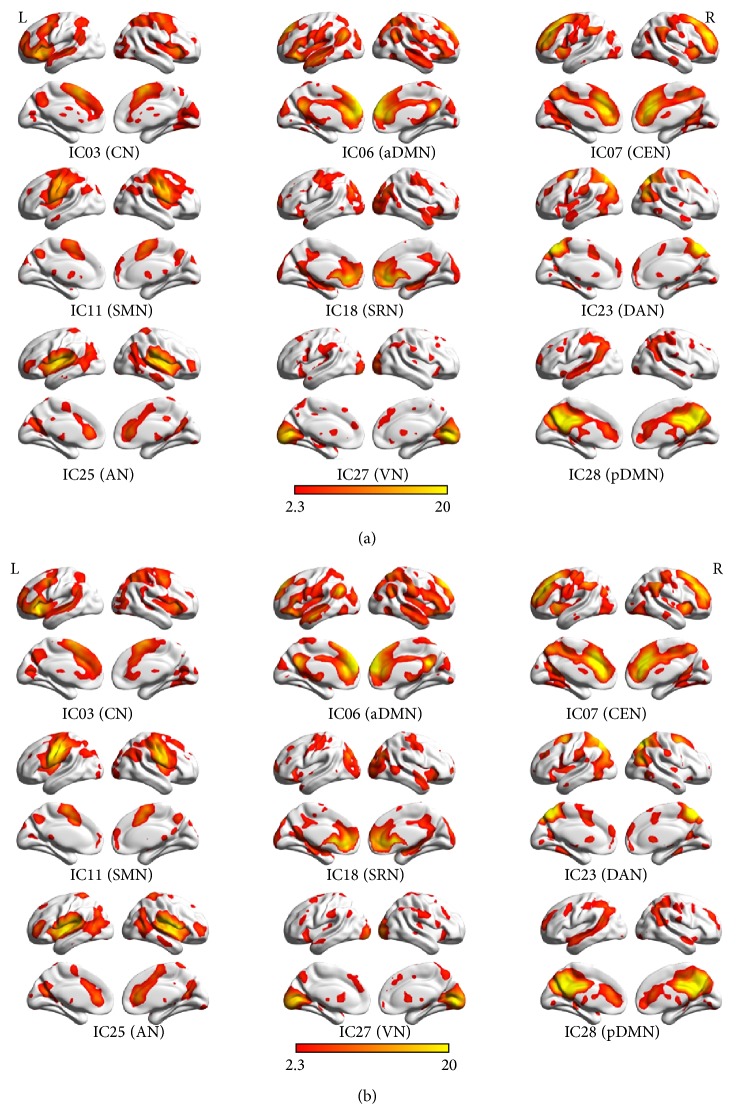
Results of one-sample *t*-test (*P* < 0.05, AlphaSim corrected) in nine RSNs of FLE (a) and HC (b), shown by BrainNet Viewer. The *T* value range is 2.3–20.

Since the DMN was split into aDMN and pDMN, nine resting-state networks (RSNs) were identified by using eight templates in all 29 ICA components for each of the 92 subjects. Correlation coefficients between the spatial templates and ICs of ICA analysis were as follows: CN (IC03), 0.297; aDMN (IC06), 0.488; CEN (IC07), 0.341; SMN (IC11), 0.755; SRN (IC18), 0.367; DAN (IC23), 0.511; AN (IC25), 0.783; VN (IC27), 0.771; pDMN (IC28), 0.383. The results of the one-sample *t*-test (*P* < 0.05, AlphaSim corrected) in the patients with FLE and HC are shown in Figure 2 by using BrainNet Viewer.

**Figure 3:**
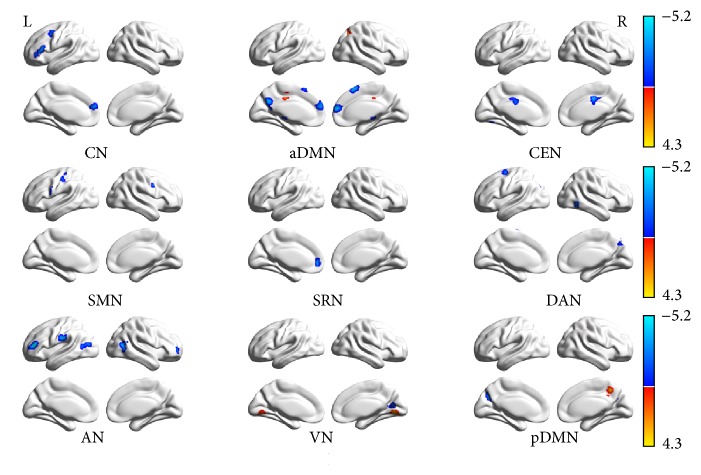
Results of the two-sample *t*-test (*P* < 0.05, AlphaSim correlated) of the RSNs between the patients with FLE and HC, shown by BrainNet Viewer. Decreased functional connectivity was observed (negative *T* value, maximum peak *T* = −5.10) in all the nine networks and increased (positive *T* value, maximum peak *T* = 4.29) functional connectivity was observed in some areas of them including aDMN, VN, and pDMN.

The two-sample *t*-test (*P* < 0.05, AlphaSim correlated) revealed the differences in functional connectivity within each RSN between the two groups. Compared with the controls, decreased functional connectivity (negative *T* value) was found in all the nine networks and increased functional connectivity (positive *T* value) was found in some of them including aDMN, VN, and pDMN. Voxels above the threshold were picked up. Within each area, the maximum *T* value and its MNI coordinate were provided. The threshold of cluster size in each RSN was also offered according to the AlphaSim correlation, as shown in Table 2 and Figure 3 by using BrainNet Viewer.

**Figure 4:**
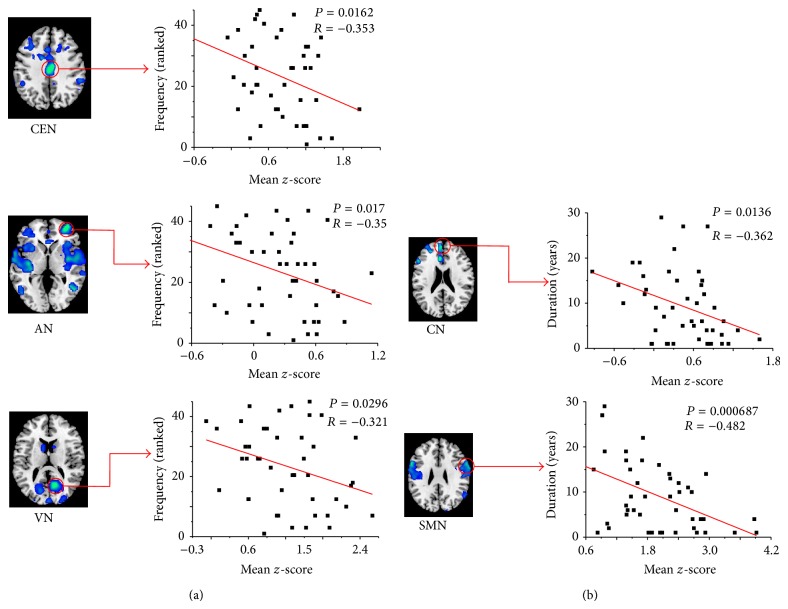
Significant negative correlations between the mean *z*-scores of ROIs and the epilepsy frequency (ranked) in CEN, AN, and VN are shown in (a). Significant negative correlations between the mean *z*-scores of ROIs and duration of epilepsy in CN and SMN are shown in (b).

Significant negative correlations were shown between the mean *z*-scores of ROIs extracted from altered RSNs and the seizure frequency in CEN (cingulate gyrus, voxels = 118, *P* = 0.0162, and *r* = −0.353), AN (Frontal_Mid_R, voxels = 41, *P* = 0.017, and *r* = −0.35), and VN (Calcarine_R, voxels = 35, *P* = 0.0296, and *r* = −0.321) in Figure 4(a). On the other hand, the significant negative correlations between the mean *z*-scores and the duration of epilepsy were found in CN (Frontal_Sup_Medial_L, voxels = 35, *P* = 0.0136, and *r* = −0.362) and SMN (Precentral_R, voxels = 56, *P* = 0.000687, and *r* = −0.482), as shown in Figure 4(b).

### 3.2. Internetwork Alterations of RSNs in FLE

**Figure 5:**
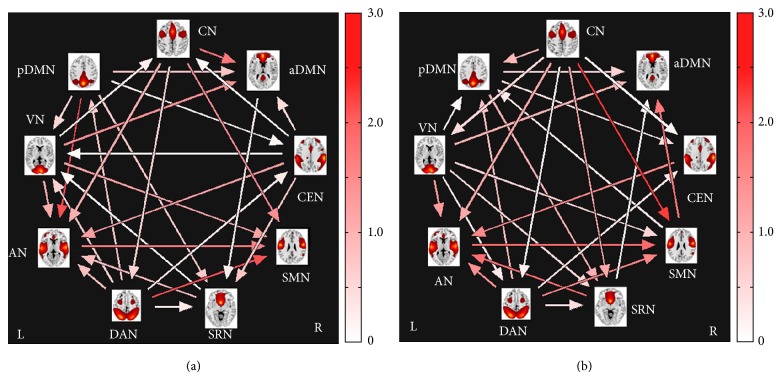
FNC results of the FLE group (a) and the HC group (b); the color bars represent time lag (0–3 s). Arrows represented a significant correlation between RSNs (*P* < 0.05, FDR corrected); 28 combinations were significant in FLE group and 27 combinations were significant in the HC group.

Significant correlation combinations were extracted for both groups separately and the results of FNC maps for each group are shown in Figure 5. It was found that 28 out of the 36 possible combinations were significant in FLE group (Figure 5(a)) and 27 out of the 36 possible combinations were significant in the HC group (Figure 5(b)). No significant difference (*P* < 0.05, FDR corrected) was found in each combination between FLE and HC group. Arrows represent a significant correlation between RSNs (*P* < 0.05, FDR corrected). For example, an arrow that connected the CN and aDMN (pointing toward the latter in the FLE group) signifies that significant correlation was obtained between them. Its pointing direction and the color of the arrow indicated the time lag from CN to aDMN.

The FNC results of the FLE group showed alterations in inter-RSNs connectivity; the combinations and the time lags were altered in the pairs of RSNs. Compared to the controls, combinations were found to be lost between aDMN/SMN, CN/SRN, CN/pDMN, and SMN/pDMN, while combinations were found to be newly added between CN/aDMN, CEN/aDMN, CEN/SRN, CEN/VN, and AN/pDMN in the FLE group. Time lags were found lower between CN/SMN but higher between DAN/SMN than in the HC group. Also observed were the directional differences in the time lags among components (i.e., from VN to CN in patients, while from CN to VN in controls).

## 4. Discussion

This study focused on the intranetwork alterations of the RSNs in the FLE. Nine RSNs were selected to be analyzed. Compared with the HC group, decreased functional connectivity was observed in all the nine RSNs; however, in some areas of RSNs, functional connectivity was increased, including aDMN (Cingulum_Mid_L, Precuneus_R), VN (lingual gyrus), and pDMN (Postcentral_R, Precuneus_R). Correlation analysis found significant negative correlation between seizure frequency and CEN (ROI in cingulate gyrus), AN (ROI in Frontal_Mid_R), and VN (ROI in Calcarine_R) and significant negative correlation between duration of epilepsy and CN (ROI in Frontal_Sup_Medial_L), SMN (ROI in Precentral_R). Compared with the HC group, alterations of combinations in the intersystem of the RSNs were determined by FNC in the FLE group, but no significant difference was found between the FLE group and the HC group.

### 4.1. Intranetwork Alterations

DMN, hypothesized to be involved in cognitive functions associated with intrinsic processing and external inputs [30], was split into the anterior areas (aDMN) and the posterior areas (pDMN) in the current study [31]. DMN involves PCC/PCUN, bilateral inferior parietal gyrus, angular gyrus, middle temporal gyrus, superior frontal gyrus, and medial frontal gyrus [25]. Danielson et al. found activity decreased in DMN in epilepsy by using a wide range of neuroimaging and electrophysiological modalities, during complex partial, generalized tonic-clonic, and absence of seizures [32]. They supported network inhibition hypothesis that active inhibition of arousal systems by seizures in certain cortical regions leads to cortical deactivation in other cortical areas. Many studies [33, 34] also presented the significant reduced functional connectivity in DMN in epilepsy patients, including the aDMN and the pDMN. They concluded that it might momentarily reduce the consciousness level and cognitive reserve. Liao et al. found functional and structural connectivity decreased in aDMN and pDMN by using functional and structural (path length and connection density derived from DTI tractography) MRI in mTLE [35], but Zhang et al. found functional connectivity increased in the pDMN, suggesting that the posterior cingulate cortex might play a compensatory role for the altered DMN in the right mTLE [36]. In the present study, decreased functional connectivity of the DMN may implicate cognitive impairment, and the increased functional connectivity of the pDMN may play a compensatory role in patients with FLE. One interpretation was that the pDMN was involved in initiation of spike and slow-wave discharges activity [15].

In the perceptual network, SMN includes pre- and postcentral gyrus, the primary sensory-motor cortices, and the supplementary motor area; AN primarily encompasses the bilateral middle and superior temporal gyrus, Heschl gyrus, and temporal pole; VN includes the inferior, middle, and superior occipital gyrus and the temporal-occipital regions along with superior parietal gyrus [25]. Zhang et al. [36] found functional connectivity decreased in perceptual networks including SMN and AN with mTLE by using fMRI. They also found functional connectivity increased in the primary visual cortex and functional connectivity decreased in the bilateral MT+ areas of the VN. They considered that the primary visual function was not impaired and there might be deficits in the high-order visual function in mTLE. Grant et al. [37] also considered that the primary visual function was not impaired in mTLE. Woodward et al. [14] and Yang et al. [38] found patients with FLE exhibited decreased functional connectivity within the motor network. Haneef et al. [39] found reduced connectivity involving areas of the sensorimotor cortex (visual, somatosensory, auditory, and primary motor) in TLE. This study's work presented consistent results in FLE with previous findings in FLE or mTLE.

In this present study, functional connectivity was found to be decreased in the CN, CEN, and DAN. It might present impairments in the three networks. CN (insula-cingulate cortices) had an important impact on cognitive control, including the anterior cingulate, the bilateral insular, and dorsolateral prefrontal cortices [25]. Key regions in CEN [40, 41] include the dorsal lateral prefrontal cortices and the posterior parietal cortices. DAN is thought to mediate goal-directed top-down processing [25] and primarily involves the middle and superior occipital gyrus, parietal gyrus, inferior, superior parietal gyrus, middle frontal gyrus, and superior frontal gyrus. Zhang et al. also found higher order cognitive processes including CN, CEN, and DAN decreased in generalized spike-and-wave discharge during absence seizure by using EEG-fMRI [36].

The functional connectivity was found decreased in the SRN in present study. SRN comprises the ventromedial prefrontal cortex (vMPFC), medial orbital prefrontal cortex (MOPFC), gyrus rectus, and pregenual anterior cingulate gyrus (PACC) [42, 43]. Qi et al. researched the impairments of SRN in minimal hepatic encephalopathy [44], and Bai et al. [45] studied the altered pattern of SRN around amnestic mild cognitive impairment, but few studies focused on SRN in epilepsy.

Decreased functional connectivity is considered to result from the disruption of neuronal connection within a functional network and is commonly used to reflect cognitive impairments in brain disorders [46]. On the contrary, increased functional connectivity is often interpreted to reflect the enhanced functionality owing to a compensatory mechanism [47].

A higher frequency of seizures and a longer duration of epilepsy were associated with progression of gray and white matter atrophy in patients with mTLE [48]. Zhang et al. [36] found the decreased functional connectivity of the mesial temporal lobe was related to the duration of epilepsy. In this study, significant negative correlations were shown between the seizure frequency and ROIs in CEN/AN/VN. This may indicate that more impairments should be related to the higher seizure frequency in CEN (ROI in cingulate gyrus), AN (ROI in Frontal_Mid_R), and VN (ROI in Calcarine_R); significant negative correlations were found between the duration of epilepsy and ROIs in CN/SMN, and longer duration may suggest more impairments in CN (ROI in Frontal_Sup_Medial_L) and SMN (ROI in Precentral_R).

### 4.2. Internetwork Alterations

In this study, combinations were found to be lost and newly added in inter-RSNs connectivity compared with controls. Moreover, alterations of the time lags were found in the pairs of RSNs. Otti et al. found lost connectivities or combinations in the patients [49] with somatoform pain disorder, indicating impairments on the system level. On the contrary, Luo et al. found no significant difference of interaction in two RSNs between TLE and HC [16]. However, in this study, no significant difference of interaction in two RSNs was found in each combination between FLE and HC group. One explanation for this phenomenon was that no obvious impairment was found on the system level in FLE. Besides, the small sample size may be another reason to worsen statistical significance. These findings could help to understand the FLE impact on the RSNs from global perspective.

*Limitation.* Study limitations included a moderate sample size and mixed medication histories. Moreover, despite the fact that the present paper focused on the functions of the resting-state networks, it was likely that the regions within each component, as well as any corresponding subregions, may have functionally distinct roles. Further studies with more imaging data and simultaneous behavioral data acquisition are needed.

## 5. Conclusion

Based on the detection of the RSNs by using ICA, 29 components were extracted by ICA, and nine RSNs were selected for study. This study explored the alterations of all the nine networks in patients with FLE. The findings of decreased and increased functional connectivity may be helpful to understanding the neuropath-physiological mechanism in patients with FLE. The decreased functional connectivity of the RSNs may implicate the impairment in patients with FLE. Increased functional connectivity of the RSNs may indicate compensatory mechanism in FLE. Five significant correlations results showed that seizure frequency and duration of epilepsy have an impact on the subregions of the RSNs in patients with FLE. Moreover, this study's work is the first study that demonstrates results of resting FNC among RSNs in patients with FLE. The results may encourage further research about the effect of FLE on the human brain.

## Figures and Tables

**Table 1:** Clinical information and demographic of patients with FLE and HC.

Groups	Men	Age (years)	Duration (years)	Seizure frequency (times per year)	Treatment
FLE (*n* = 46)	21	26 ± 4.59 (19–35)	9.2 ± 7.82 (1–27)	59.66 ± 166.67 (0.42–912)	Carbamazepine: 11, topiramate: 5, oxcarbazepine: 3, phenobarbital: 6, sodium valproate: 16, no treatment: 5

HC (*n* = 46)	22	25.3 ± 5.33 (20–39)	/	/	/

**Table 2:** Group comparisons of the nine RSNs between the patients with FLE and HC.

Brain regions	Coordinates	*T* value (peak)	Voxels
	*x*, *y*, *z* (MNI)		
CN (cluster size > 33)			
Frontal_Inf_Tri_L	−51 24 18	−3.55	96
Frontal_Sup_Medial_L	−3 57 21	−3.27	35
Precentral_L	−39 3 48	−3.29	34
aDMN (cluster size > 36)			
Frontal_Sup_Medial_L	9 57 15	−4.54	172
Superior frontal gyrus	3 18 57	−3.71	71
Precuneus_L	−3 −63 33	−3.90	67
Angular_L	−39 −57 30	−5.10	48
Cingulum_Mid_L	−6 −24 42	4.19	38
Precuneus_R	21 −63 42	3.61	37
CEN (cluster size > 43)			
Cingulate gyrus	6 −12 39	−3.87	118
SMN (cluster size > 34)			
Precentral_L	−60 9 24	−3.19	56
Precentral_R	60 9 33	−2.79	56
Postcentral_L	−42 −24 66	−3.66	52
SRN (cluster size > 26)			
Medial frontal gyrus	−3 51 0	−3.66	69
DAN (cluster size > 35)			
Temporal_Inf_R	48 −60 −6	−4.62	37
Frontal_Sup_L	−18 −3 63	−4.04	44
Occipital_Mid_L	−24 −72 30	−3.34	42
Precuneus_R	0 −78 51	−3.82	49
AN (cluster size > 36)			
Frontal_Inf_Tri_L	−39 39 12	−4.97	69
Frontal_Mid_R	36 57 0	−4.32	41
Temporal_Mid_R	42 −60 12	−3.63	84
Temporal_Mid_L	−42 −63 9	−3.60	50
Postcentral_L	−63 −21 27	−3.87	104
VN (cluster size > 34)			
Lingual gyrus	15 −69 0	4.29	186
Calcarine_R	12 −63 15	−3.64	35
pDMN (cluster size > 36)			
Postcentral_R	27 −30 42	4.07	68
Precuneus_R	18 −48 45	3.71	70
Precuneus_L	−15 −66 33	−4.41	52
Precuneus_R	21 −63 30	−3.55	41

Note: *P* < 0.05, AlphaSim corrected.
